# Retraction: The Uptake of Apoptotic Cells Drives *Coxiella burnetii* Replication and Macrophage Polarization: A Model for Q Fever Endocarditis

**DOI:** 10.1371/journal.ppat.1014210

**Published:** 2026-05-12

**Authors:** The PLOS Pathogens Editors

The *PLOS Pathogens* Editors retract this article [1, 2] due to concerns about compliance with the PLOS Human Subjects Research policy.

Following the publication of the article and Expression of Concern [1,2], PLOS investigated concerns pertaining to the reported ethical approval and the article’s adherence to *PLOS Pathogens*’ research ethics policies.

Specifically, the Materials and Methods section in [1] states that the study used blood samples collected from patients presenting with valvulopathy, acute Q fever, and Q fever endocarditis, as well as healthy controls, and that the study was approved by the Ethics Committee of the Université de la Méditerranée. The study does not report when or where the samples were collected, nor does it report an ethics approval reference number.

The authors did not respond to the journal’s request for additional information and ethics approval documentation.

A representative of the Aix-Marseille Université Ethics Committee stated that in France, the law requires ethics approval documents to be kept for 15 years following the end date of the study, and that the institute is unable to provide the requested documentation after the considerable time that has passed since the study was completed.

In the absence of the requested documentation, PLOS is unable to confirm the article’s compliance with the PLOS Human Subjects Research policy.

JLM responded but expressed neither agreement nor disagreement with the editorial decision. MB, EG, CC, and DR either did not respond directly or could not be reached.
